# Association of the atherogenic index of plasma combined with obesity indices with cardiovascular disease and mortality

**DOI:** 10.1186/s12944-026-02932-3

**Published:** 2026-03-19

**Authors:** Xinran Wang, Sirun Qin, Hong Xiang, Ye Tao, Shuhua Chen, Hongwei Lu

**Affiliations:** 1 Department of Cardiology, The Third Xiangya Hospital, Central South University, Changsha, China; 2 Department of Nephrology, The Third Xiangya Hospital, Central South University, Changsha, China; 3 Department of Cardiology, Renji Hospital, School of Medicine, Shanghai Jiao Tong University, Shanghai, China; 4 Center for Experimental Medicine, The Third Xiangya Hospital, Central South University, Changsha, China; 5 Department of Biochemistry, School of Life Sciences, Central South University, Changsha, China

**Keywords:** Atherogenic index of plasma, Obesity indices, All-cause mortality, Cardiovascular disease mortality, Cardiovascular disease

## Abstract

**Background:**

Both the atherogenic index of plasma (AIP) and obesity have been implicated in cardiovascular disease (CVD) risk. This study aimed to investigate the associations of AIP and AIP-obesity indices (AIP-WC, AIP-BMI, AIP-WHtR, AIP-WWI, and AIP-BRI) with CVD and mortality.

**Methods:**

This study enrolled 21,944 individuals from the National Health and Nutrition Examination Survey (NHANES) fielded during the years 1999–2018. Cox and logistic regression modeling, along with restricted cubic splines (RCS), served to assess the associations of AIP and AIP-obesity indices with all-cause mortality, CVD mortality, and CVD prevalence. The incremental predictive value of AIP and AIP-obesity indices was further assessed.

**Results:**

Throughout an average follow-up period of 116 months, 3,326 all-cause deaths were documented, among which 1,042 were CVD deaths. AIP, AIP-WC, AIP-BMI, AIP-WHtR, AIP-WWI, and AIP-BRI were significantly associated with all-cause mortality, CVD mortality, and CVD prevalence. In the fully adjusted model, relative to the lowest-tertile participants, the highest-tertile participants of these indices had an elevated risk of 17.7–20.3% for all-cause mortality, an elevated risk of 30.3–33.8% for CVD mortality, and a 103.7–122.2% increase in the odds of CVD. RCS analyses indicated non-linear associations of AIP, AIP-WC, AIP-BMI, AIP-WHtR, AIP-WWI, and AIP-BRI with all-cause mortality and CVD prevalence, whereas linear associations were observed with CVD mortality. Incremental predictive value analyses showed that compared with AIP alone, AIP-WC, AIP-BMI, AIP-WHtR, AIP-WWI, and AIP-BRI provided superior risk reclassification and discrimination across all outcomes.

**Conclusions:**

AIP and AIP-obesity indices were significantly associated with CVD and mortality risk. AIP combined with obesity-related indices, particularly BRI, provides additional value for the stratification of CVD and mortality risk.

**Supplementary Information:**

The online version contains supplementary material available at 10.1186/s12944-026-02932-3.

## Background

Cardiovascular disease (CVD) remains the primary contributor to mortality globally, resulting in substantial health loss and creating heavy economic liabilities for healthcare systems [1–3]. Although notable advances have been realized in CVD-related prevention, diagnosis, and treatment efforts lately, its global health burden remains on the rise [1, 4, 5]. Consequently, systematically identifying and integrating CVD-related risk determinants is essential for formulating more efficient approaches to disease prevention and clinical management.

The atherogenic index of plasma (AIP) is an emerging metabolic biomarker that reflects the equilibrium between anti-atherogenic and pro-atherogenic lipoproteins [6]. Elevated AIP represents an abnormal lipid profile that promotes endothelial dysfunction and foam cell formation in atherosclerotic lesions, exhibiting a significant correlation with the severity of atherosclerosis [6–9]. Furthermore, AIP is also recognized as a pivotal predictor of cardiovascular risk [10, 11]. Obesity has spread to epidemic proportions, with the number of affected individuals steadily increasing [12]. Obesity has long been recognized as an important risk factor for CVD [13–15]. One key mechanism is the presence of chronic, low-grade inflammation in visceral adipose tissue. Inflammatory activity and oxidative stress within adipose tissue reduce adiponectin production and increase the secretion of pro-inflammatory adipokines and cytokines, promoting vascular stiffness and ultimately impairing cardiac diastolic function [16, 17].

Both atherosclerosis and obesity are significant contributing factors to the development and progression of CVD. Integrating these two factors is of great significance for the assessment of CVD-related risk. AIP is an established and effective metric for evaluating atherosclerosis severity, while obesity-associated indices—such as waist circumference (WC), body mass index (BMI), waist-to-height ratio (WHtR), weight-adjusted waist index (WWI), and body roundness index (BRI)—can provide a comprehensive evaluation of obesity status [6, 18–20]. Therefore, it is necessary to combine AIP with obesity-associated indices to enable a more comprehensive assessment of CVD-related risk in the population. However, research on the combined use of AIP and obesity-associated indices in relation to CVD and mortality remains very limited. This study harnessed the large-volume dataset from the National Health and Nutrition Examination Survey (NHANES) to examine the associations of AIP and AIP-obesity indices with CVD and mortality.

## Methods

### Study population

**Fig. 1:**
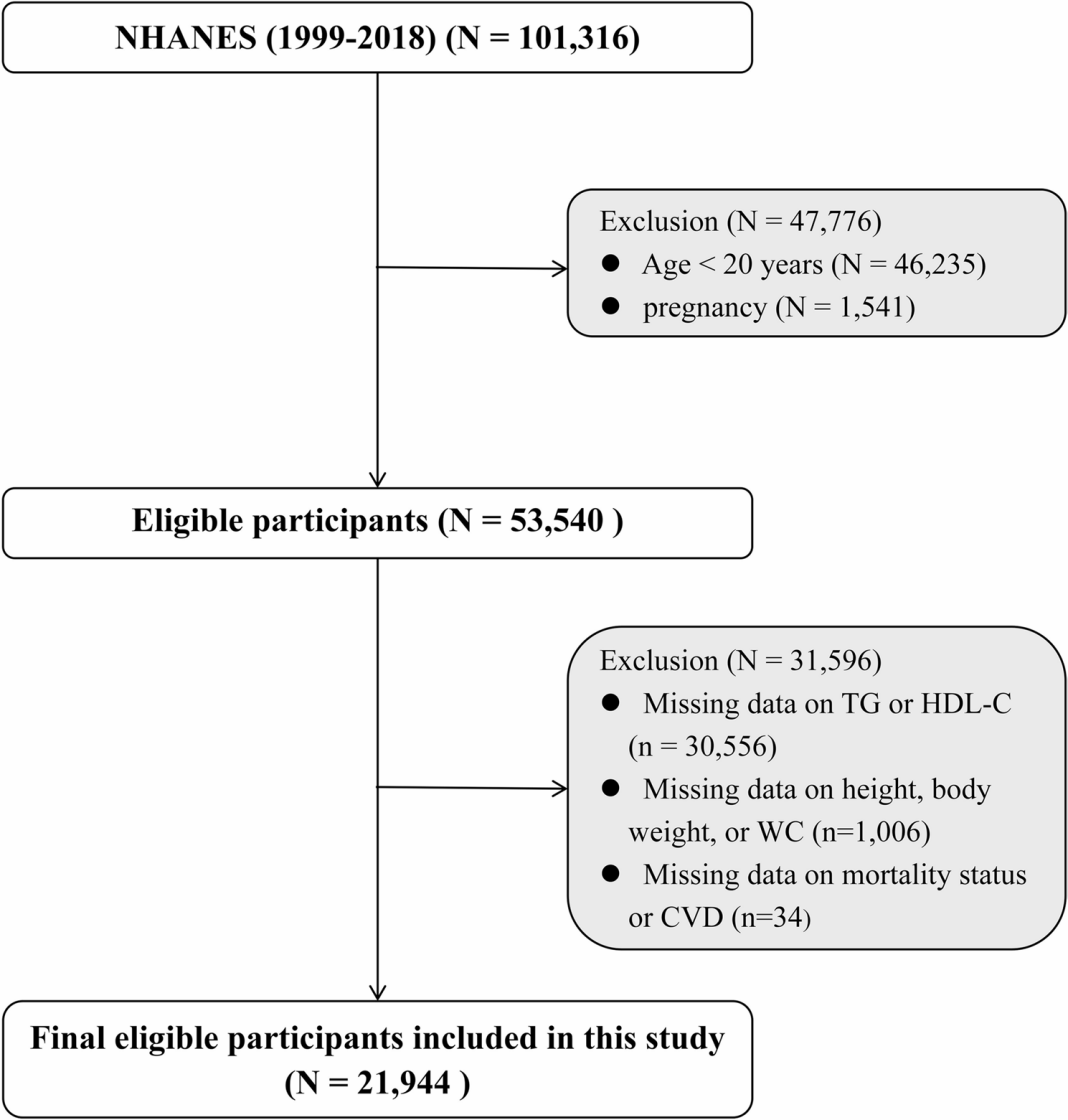
Flow chart of study participants

Data from the 1999–2018 NHANES were employed for this study, initially including 101,316 participants. Exclusion criteria were: (1) age < 20 years (*n* = 46,235); (2) pregnancy (*n* = 1,541); (3) missing AIP data (*n* = 30,556); (4) missing weight, height, or WC measurements (*n* = 1,006); or (5) incomplete information on mortality or CVD (*n* = 34). After applying these exclusion criteria, the final analysis incorporated 21,944 eligible participants (Fig. 1).

### Data collection

This study extracted demographic data, physical examination findings, laboratory measurements, and medical history for participants. Demographic variables included sex, age, race, marital status, education, family poverty-to-income ratio (PIR), smoking status, and alcohol consumption. Physical examination parameters comprised height, weight, WC, and blood pressure. Laboratory parameters included high-density lipoprotein cholesterol (HDL-C), triglycerides (TG), total cholesterol (TC), glycated hemoglobin (HbA1c), and fasting blood glucose (FBG). Information on medical history included hypertension and diabetes. Participants were classified as having hypertension if they reported a previous diagnosis, were receiving antihypertensive medications, or had a systolic blood pressure ≥ 140 mmHg or diastolic blood pressure ≥ 90 mmHg [21]. Participants were identified as having diabetes if they reported a previous diagnosis, were using glucose-lowering medications, or had an FBG ≥ 126 mg/dL or HbA1c ≥ 6.5% [21, 22].

### Exposure variables and outcome definitions

The exposure variables consisted of AIP and AIP-obesity indices (AIP-WC, AIP-BMI, AIP-WHtR, AIP-WWI, and AIP-BRI). All indices were computed using the following formulas [18, 23, 24]:

$$BMI=\frac{\mathrm{Weigth}(kg)}{\mathrm{Height}(m)^{2}}$$

$$WHTR=\frac{\mathrm{WC}(cm)}{\mathrm{Height}(cm)}$$

$$WWI=\frac{\mathrm{WC}(cm)}{\sqrt{\mathrm{Weight}(kg)}}$$

$$BRI=364.2-365.5^{\ast}\sqrt{1-\frac{[\mathrm{WC}(m)/2\pi]^{2}}{[0.5^{\ast}\mathrm{Height}(m)]^{2}}}$$

$$AIP=\mathrm{log}_{10}\left(\frac{\mathrm{TG}[mg/dL]}{\mathrm{HDL-C}[mg/dL]}\right)$$

$$AIP-WC=AIP^{\ast}WC$$

$$AIP-BMI=AIP^{\ast}BMI$$

$$AIP-WHTR=AIP^{\ast}WHTR$$

$$AIP-BRI=AIP^{\ast}BRI$$

$$AIP-WWI=AIP^{*}WWI$$

Mortality outcomes comprised all-cause and cardiovascular death. Information on deaths in NHANES was obtained from the National Death Index through linkage with death certificates. All-cause mortality was defined as death from any cause, whereas cardiovascular mortality (I00–I09, I11, I13, I20–I51, I60–I69) included deaths due to heart disease or cerebrovascular disease [25, 26].

CVD outcomes were based on physician-diagnosed conditions reported by participants, including coronary heart disease, heart attack, angina pectoris, stroke, or congestive heart failure [25].

### Statistical analysis

Given that NHANES employed a multi-stage stratified probability sampling scheme, all statistical analyses accounted for sample weights, strata, and clusters to address potential biases. Missing data were addressed using multiple imputation via chained equations. The exposure variables were categorized into tertiles (T1–T3). Continuous variables were presented as weighted means ± standard errors (SE), and categorical variables were expressed as unweighted counts with weighted percentages. Group differences were assessed using the chi-square test, t-test or Mann-Whitney U test.

Kaplan–Meier curves were used to evaluate differences in all-cause and CVD mortality across tertiles of AIP and AIP-obesity indices. Associations of AIP and AIP-obesity indices with all-cause and CVD mortality were assessed via Cox proportional hazards regression, while associations with CVD prevalence were examined using logistic regression. Three models were constructed: Model 1 was unadjusted; Model 2 was adjusted for age, sex, and race; Model 3 included the variables in Model 2 and additionally adjusted for marital status, education, smoking status, alcohol use, PIR, and TC. The variance inflation factor (VIF) was applied to detect multicollinearity among exposure variables and covariates. Schoenfeld residuals were employed to assess the proportional hazards assumption. Restricted cubic spline (RCS) regression with four knots placed at the 5th, 35th, 65th, and 95th percentiles was applied to explore potential linear or nonlinear dose–response relationships of AIP and AIP-obesity indices with all-cause mortality, CVD mortality, and CVD prevalence. Subgroup analyses were conducted to examine the associations across key demographic and clinical subgroups. Incremental predictive value analyses were used to investigate the ability of AIP and AIP-obesity indices to improve risk reclassification and discrimination for CVD and mortality. Three sensitivity analyses were carried out: first, participants who died early in follow-up (within two years) were excluded to minimize reverse causality; second, those with CVD and cancer were excluded to reduce the confounding effect of severe underlying diseases on mortality risk; finally, participants whose covariates were imputed were excluded to assess the robustness of the multiple imputation approach.

All statistical analyses were implemented via R software (version 4.5.1), and two-tailed *P* values < 0.05 were considered statistically significant.

## Results

### Baseline characteristics of participants

A total of 21,944 participants were included in this study, of whom 49.42% were male and 69.11% were Non-Hispanic White. The mean age of participants was 47.39 years. Compared with participants who did not experience CVD mortality, those in the CVD death group were older and included a higher proportion of males, Non-Hispanic Whites, unmarried or non-cohabiting individuals, and individuals with lower educational attainment, as well as a higher prevalence of hypertension and diabetes. Additionally, this group exhibited greater WC, FPG, HbA1c, TG, AIP, AIP-WC, AIP-BMI, AIP-WHtR, AIP-WWI, and AIP-BRI, while height was lower (Table 1). Tables S1 and S2 summarize the baseline characteristics of participants who experienced all-cause mortality and individuals diagnosed with CVD, respectively.

**Table 1:** Baseline characteristics according to CVD mortality

Variable	Total (*n* = 21944)	CVD mortality		*P*
		No (*n* = 20902)	Yes (*n* = 1042)	
Sex, n (%)				**< 0.001**
Male	10,922 (49.42)	10,309 (49.19)	613 (56.28)	
Female	11,022 (50.58)	10,593 (50.81)	429 (43.72)	
Age, year	47.39 (0.22)	46.69 (0.21)	68.34 (0.62)	**< 0.001**
Race, n (%)				**< 0.001**
Mexican American	3886 (8.18)	3752 (8.33)	134 (3.72)	
Non-Hispanic White	9820 (69.11)	9192 (68.83)	628 (77.49)	
Non-Hispanic Black	4362 (10.42)	4157 (10.39)	205 (11.38)	
Other Race	3876 (12.29)	3801 (12.45)	75 (7.41)	
Marital status, n (%)				**< 0.001**
Married or living with partner	13,418 (64.96)	12,860 (65.26)	558 (56.08)	
Separated/divorced/widowed	4803 (18.19)	4385 (17.55)	418 (37.44)	
Never	3723 (16.85)	3657 (17.20)	66 (6.48)	
Education, n (%)				**< 0.001**
Below high school	5976 (17.59)	5572 (17.19)	404 (29.58)	
High school or equivalent	5037 (24.08)	4773 (23.91)	264 (29.31)	
College or above	10,931 (58.32)	10,557 (58.90)	374 (41.10)	
Smoking status, n (%)				**< 0.001**
Never	11,733 (52.74)	11,275 (53.04)	458 (43.81)	
Former	5566 (25.71)	5161 (25.31)	405 (37.68)	
Current	4645 (21.55)	4466 (21.65)	179 (18.51)	
Alcohol use, n (%)				**< 0.001**
Never	6472 (25.27)	6079 (24.92)	393 (35.83)	
Ever	15,472 (74.73)	14,823 (75.08)	649 (64.17)	
PIR, n (%)				**< 0.001**
< 1.3	6633 (21.07)	6287 (20.93)	346 (25.47)	
1.3–3.5	8466 (36.50)	8005 (36.19)	461 (45.86)	
> 3.5	6845 (42.43)	6610 (42.89)	235 (28.67)	
Height, cm	169.02 (0.09)	169.09 (0.09)	167.10 (0.41)	**< 0.001**
BMI, kg/m^2^	28.68 (0.08)	28.67 (0.08)	29.00 (0.28)	0.262
WC, cm	98.39 (0.20)	98.24 (0.20)	102.92 (0.66)	**< 0.001**
Hypertension, n (%)				**< 0.001**
No	12,605 (62.80)	12,366 (64.04)	239 (25.59)	
Yes	9339 (37.20)	8536 (35.96)	803 (74.41)	
Diabetes, n (%)				**< 0.001**
No	17,695 (85.62)	17,045 (86.35)	650 (63.76)	
Yes	4249 (14.38)	3857 (13.65)	392 (36.24)	
FPG, mg/dL	105.38 (0.30)	104.85 (0.31)	120.99 (1.77)	**< 0.001**
HbA1c, %	5.58 (0.01)	5.56 (0.01)	6.09 (0.05)	**< 0.001**
TC, mg/dL	195.38 (0.46)	195.33 (0.46)	196.68 (1.72)	0.409
TG, mg/dL	132.38 (1.22)	131.66 (1.25)	153.86 (3.71)	**< 0.001**
HDL-C, mg/dL	53.61 (0.19)	53.62 (0.20)	53.14 (0.56)	0.429
AIP	0.33 (0.00)	0.32 (0.00)	0.42 (0.01)	**< 0.001**
AIP-WC	34.24 (0.43)	33.89 (0.44)	44.73 (1.35)	**< 0.001**
AIP-BMI	10.04 (0.13)	9.95 (0.13)	12.72 (0.40)	**< 0.001**
AIP-WHTR	0.20 (0.00)	0.20 (0.00)	0.27 (0.01)	**< 0.001**
AIP-BRI	1.94 (0.03)	1.92 (0.03)	2.68 (0.09)	**< 0.001**
AIP-WWI	3.64 (0.04)	3.60 (0.05)	4.85 (0.14)	**< 0.001**
AIP tertile groups, n (%)				**< 0.001**
1	7101 (32.98)	6869 (33.38)	232 (21.03)	
2	7474 (33.97)	7101 (33.91)	373 (35.83)	
3	7369 (33.05)	6932 (32.71)	437 (43.13)	
AIP-WC tertile groups, n (%)				**< 0.001**
1	7070 (33.00)	6846 (33.42)	224 (20.39)	
2	7549 (34.00)	7163 (33.91)	386 (36.63)	
3	7325 (33.00)	6893 (32.67)	432 (42.98)	
AIP-BMI tertile groups, n (%)				**< 0.001**
1	7059 (33.00)	6828 (33.39)	231 (21.27)	
2	7549 (34.00)	7152 (33.89)	397 (37.12)	
3	7336 (33.00)	6922 (32.71)	414 (41.61)	
AIP-WHTR tertile groups, n (%)				**< 0.001**
1	7050 (32.99)	6828 (33.42)	222 (20.40)	
2	7472 (34.00)	7095 (33.94)	377 (35.89)	
3	7422 (33.00)	6979 (32.64)	443 (43.71)	
AIP-BRI tertile groups, n (%)				**< 0.001**
1	6960 (32.99)	6749 (33.44)	211 (19.68)	
2	7423 (34.00)	7054 (33.98)	369 (34.79)	
3	7561 (33.00)	7099 (32.58)	462 (45.53)	
AIP-WWI tertile groups, n (%)				**< 0.001**
1	7069 (33.00)	6851 (33.44)	218 (19.82)	
2	7428 (34.00)	7055 (33.94)	373 (35.61)	
3	7447 (33.00)	6996 (32.62)	451 (44.57)	

Continuous variables were expressed as mean (SE), and group differences were assessed using the t-test or Mann-Whitney U test. Categorical variables were expressed as counts (percentages), and group differences were assessed using the chi-square test

Bold values indicate *p*-values < 0.05

*Abbreviations*: *CVD* Cardiovascular disease, *PIR* Income-to-poverty ratio, *BMI* Body mass index, *WC* Waist circumference, FBG Fasting blood glucose, *HbA1c* Glycated hemoglobin, *TC* Total cholesterol, *TG* Triglycerides, *HDL-C* High-density lipoprotein cholesterol, *AIP* Atherogenic index of plasma, *WHtR* Waist-to-height ratio, *BRI* Body roundness index, *WWI* Weight-adjusted waist index, *SE* Standard errors

### Associations of AIP and AIP-obesity indices with CVD and mortality

Throughout an average follow-up period of 116 months, 3,326 all-cause deaths were documented, among which 1,042 were CVD deaths. Figure S1 displays Kaplan–Meier survival curves that characterize the probability of overall and CVD-specific survival across tertiles of AIP and AIP-obesity indices. Participants in higher tertiles showed lower overall and CVD-specific survival.

Tables 2 and 3 show the associations of AIP and AIP-obesity indices with all-cause mortality, CVD mortality, and CVD prevalence. In univariable Cox regression analyses (Model 1), AIP, AIP-WC, AIP-BMI, AIP-WHtR, AIP-WWI, and AIP-BRI were all significantly associated with both all-cause and CVD mortality. These associations remained statistically significant after full adjustment for confounders (Model 3). Furthermore, in the fully adjusted models (Model 3), compared with the lowest tertile (T1) group, individuals in the highest tertile (T3) of AIP, AIP-WC, AIP-BMI, AIP-WHtR, AIP-WWI, and AIP-BRI showed significantly higher risk of all-cause mortality (HR 1.177–1.203) and CVD mortality (HR 1.303–1.338). Similarly, univariable logistic regression analyses (Model 1) indicated that AIP, AIP-WC, AIP-BMI, AIP-WHtR, AIP-WWI, and AIP-BRI were significantly associated with CVD. These associations remained statistically significant after full adjustment for confounders (Model 3). In fully adjusted models, compared with the T1 group, individuals in the T2 and T3 groups of AIP, AIP-WC, AIP-BMI, AIP-WHtR, AIP-WWI, and AIP-BRI had increased odds of CVD (T2 OR 1.249–1.331; T3 OR 2.037–2.222). Schoenfeld residual analysis showed that the proportional hazards assumptions of the Cox model were not violated (all *P* > 0.05), and VIF tests indicated no substantial multicollinearity among the covariates (all VIF < 5) (Tables S3 and S4).

**Table 2:** Associations of AIP and AIP-obesity indices with all-cause mortality and CVD mortality

Variables	Model1	Model2	Model3
	HR (95%CI)	HR (95%CI)	HR (95%CI)
All-cause mortality			
AIP	1.712 (1.492–1.965)	1.482 (1.264–1.738)	1.366 (1.174–1.589)
AIP-WC	1.006 (1.004–1.007)	1.004 (1.003–1.006)	1.003 (1.002–1.005)
AIP-BMI	1.015 (1.011–1.019)	1.013 (1.008–1.018)	1.011 (1.007–1.016)
AIP-WHTR	2.817 (2.269–3.497)	2.011 (1.573–2.570)	1.775 (1.410–2.234)
AIP-BRI	1.113 (1.093–1.134)	1.074 (1.051–1.098)	1.062 (1.041–1.083)
AIP-WWI	1.064 (1.050–1.077)	1.039 (1.025–1.054)	1.030 (1.017–1.044)
AIP tertile groups			
T1	Reference	Reference	Reference
T2	1.374 (1.236–1.528)	1.110 (0.997–1.236)	1.037 (0.934–1.152)
T3	1.631 (1.449–1.836)	1.304 (1.164–1.461)	1.203 (1.082–1.338)
AIP-WC tertile groups			
T1	Reference	Reference	Reference
T2	1.373 (1.222–1.543)	1.079 (0.958–1.215)	1.023 (0.912–1.147)
T3	1.709 (1.522–1.920)	1.273 (1.131–1.434)	1.180 (1.054–1.321)
AIP-BMI tertile groups			
T1	Reference	Reference	Reference
T2	1.381 (1.231–1.550)	1.067 (0.947–1.203)	1.009 (0.898–1.133)
T3	1.588 (1.416–1.780)	1.253 (1.119–1.402)	1.177 (1.057–1.311)
AIP-WHTR tertile groups			
T1	Reference	Reference	Reference
T2	1.354 (1.205–1.522)	1.067 (0.953–1.194)	1.005 (0.903–1.118)
T3	1.752 (1.561–1.966)	1.280 (1.140–1.437)	1.185 (1.061–1.324)
AIP-BRI tertile groups			
T1	Reference	Reference	Reference
T2	1.437 (1.275–1.620)	1.051 (0.929–1.188)	0.996 (0.885–1.119)
T3	1.924 (1.719–2.153)	1.271 (1.134–1.425)	1.184 (1.063–1.318)
AIP-WWI tertile groups			
T1	Reference	Reference	Reference
T2	1.348 (1.200–1.514)	1.083 (0.958–1.225)	1.012 (0.899–1.140)
T3	1.779 (1.578–2.005)	1.304 (1.154–1.473)	1.192 (1.060–1.341)
CVD mortality			
AIP	1.777 (1.428–2.211)	1.602 (1.222–2.101)	1.523 (1.166–1.989)
AIP-WC	1.006 (1.004–1.008)	1.005 (1.003–1.008)	1.005 (1.002–1.007)
AIP-BMI	1.018 (1.011–1.024)	1.019 (1.010–1.027)	1.017 (1.009–1.026)
AIP-WHTR	3.198 (2.275–4.494)	2.468 (1.640–3.714)	2.268 (1.521–3.382)
AIP-BRI	1.131 (1.100–1.164)	1.102 (1.064–1.142)	1.092 (1.055–1.130)
AIP-WWI	1.069 (1.048–1.090)	1.047 (1.023–1.071)	1.041 (1.018–1.065)
AIP tertile groups			
T1	Reference	Reference	Reference
T2	1.483 (1.226–1.794)	1.171 (0.970–1.414)	1.114 (0.925–1.340)
T3	1.728 (1.392–2.146)	1.390 (1.124–1.719)	1.317 (1.071–1.618)
AIP-WC tertile groups			
T1	Reference	Reference	Reference
T2	1.581 (1.283–1.950)	1.213 (0.991–1.484)	1.172 (0.960–1.432)
T3	1.854 (1.507–2.281)	1.379 (1.124–1.691)	1.303 (1.068–1.590)
AIP-BMI tertile groups			
T1	Reference	Reference	Reference
T2	1.533 (1.254–1.874)	1.150 (0.944–1.400)	1.108 (0.913–1.344)
T3	1.716 (1.394–2.113)	1.370 (1.122–1.673)	1.304 (1.072–1.586)
AIP-WHTR tertile groups			
T1	Reference	Reference	Reference
T2	1.540 (1.252–1.895)	1.182 (0.968–1.445)	1.139 (0.935–1.387)
T3	1.897 (1.541–2.334)	1.386 (1.127–1.704)	1.309 (1.067–1.605)
AIP-BRI tertile groups			
T1	Reference	Reference	Reference
T2	1.560 (1.252–1.945)	1.106 (0.894–1.368)	1.073 (0.870–1.323)
T3	2.151 (1.760–2.629)	1.411 (1.158–1.720)	1.336 (1.099–1.626)
AIP-WWI tertile groups			
T1	Reference	Reference	Reference
T2	1.562 (1.279–1.909)	1.224 (1.003–1.494)	1.164 (0.958–1.415)
T3	1.952 (1.578–2.416)	1.424 (1.147–1.769)	1.338 (1.081–1.657)

Associations of AIP and AIP-obesity indices with all-cause and CVD mortality were assessed via Cox proportional hazards regression

Model 1 was unadjusted; Model 2 was adjusted for age, sex, and race; Model 3 was adjusted for age, sex, race, marital status, education, smoking status, alcohol use, income-to-poverty ratio, and total cholesterol

*Abbreviations*: *HR* Hazard ratio, *CI* Confidence interval, *CVD* Cardiovascular disease, *AIP* Atherogenic index of plasma, *BMI* Body mass index, *WC* Waist circumference, *WHtR* Waist-to-height ratio, *BRI* Body roundness index, *WWI* Weight-adjusted waist index

**Table 3:** Associations of AIP and AIP-obesity indices with CVD prevalence

Variables	Model1	Model2	Model3
	OR (95%CI)	OR (95%CI)	OR (95%CI)
CVD			
AIP	2.250 (1.884–2.687)	2.317 (1.884–2.850)	2.608 (2.073–3.282)
AIP-WC	1.009 (1.007–1.010)	1.009 (1.007–1.011)	1.010 (1.008–1.012)
AIP-BMI	1.026 (1.021–1.032)	1.030 (1.024–1.037)	1.034 (1.027–1.041)
AIP-WHTR	4.594 (3.460–6.099)	4.434 (3.217–6.112)	5.077 (3.606–7.147)
AIP-BRI	1.165 (1.138–1.194)	1.157 (1.126–1.189)	1.161 (1.130–1.194)
AIP-WWI	1.089 (1.071–1.107)	1.081 (1.062–1.101)	1.090 (1.069–1.112)
AIP tertile groups			
T1	Reference	Reference	Reference
T2	1.500 (1.300–1.731)	1.347 (1.160–1.564)	1.296 (1.115–1.507)
T3	2.103 (1.809–2.446)	1.997 (1.701–2.344)	2.064 (1.750–2.434)
AIP-WC tertile groups			
T1	Reference	Reference	Reference
T2	1.496 (1.287–1.740)	1.333 (1.141–1.557)	1.302 (1.112–1.524)
T3	2.304 (1.977–2.684)	2.077 (1.766–2.443)	2.108 (1.786–2.487)
AIP-BMI tertile groups			
T1	Reference	Reference	Reference
T2	1.505 (1.307–1.732)	1.318 (1.143–1.520)	1.297 (1.120–1.502)
T3	2.217 (1.894–2.596)	2.118 (1.794–2.500)	2.187 (1.851–2.585)
AIP-WHTR tertile groups			
T1	Reference	Reference	Reference
T2	1.489 (1.278–1.735)	1.322 (1.130–1.545)	1.286 (1.097–1.507)
T3	2.389 (2.048–2.786)	2.126 (1.811–2.497)	2.176 (1.848–2.562)
AIP-BRI tertile groups			
T1	Reference	Reference	Reference
T2	1.495 (1.285–1.740)	1.260 (1.080–1.470)	1.249 (1.070–1.458)
T3	2.688 (2.305–3.135)	2.196 (1.874–2.574)	2.222 (1.886–2.618)
AIP-WWI tertile groups			
T1	Reference	Reference	Reference
T2	1.522 (1.309–1.769)	1.374 (1.176–1.607)	1.331 (1.136–1.558)
T3	2.292 (1.969–2.668)	2.009 (1.709–2.362)	2.037 (1.727–2.402)

Associations of AIP and AIP-obesity indices with CVD prevalence were assessed via logistic regression

Model 1 was unadjusted; Model 2 was adjusted for age, sex, and race; Model 3 was adjusted for age, sex, race, marital status, education, smoking status, alcohol use, income-to-poverty ratio, and total cholesterol

*Abbreviations*: *OR* Odds ratio, *CI* Confidence interval, *CVD* Cardiovascular disease, *AIP* Atherogenic index of plasma, *BMI* Body mass index, *WC* Waist circumference, *WHtR* Waist-to-height ratio, *BRI* Body roundness index, *WWI* weight-adjusted waist index

### Dose–response relationship of AIP and AIP-obesity indices with CVD and mortality

**Fig. 2:**
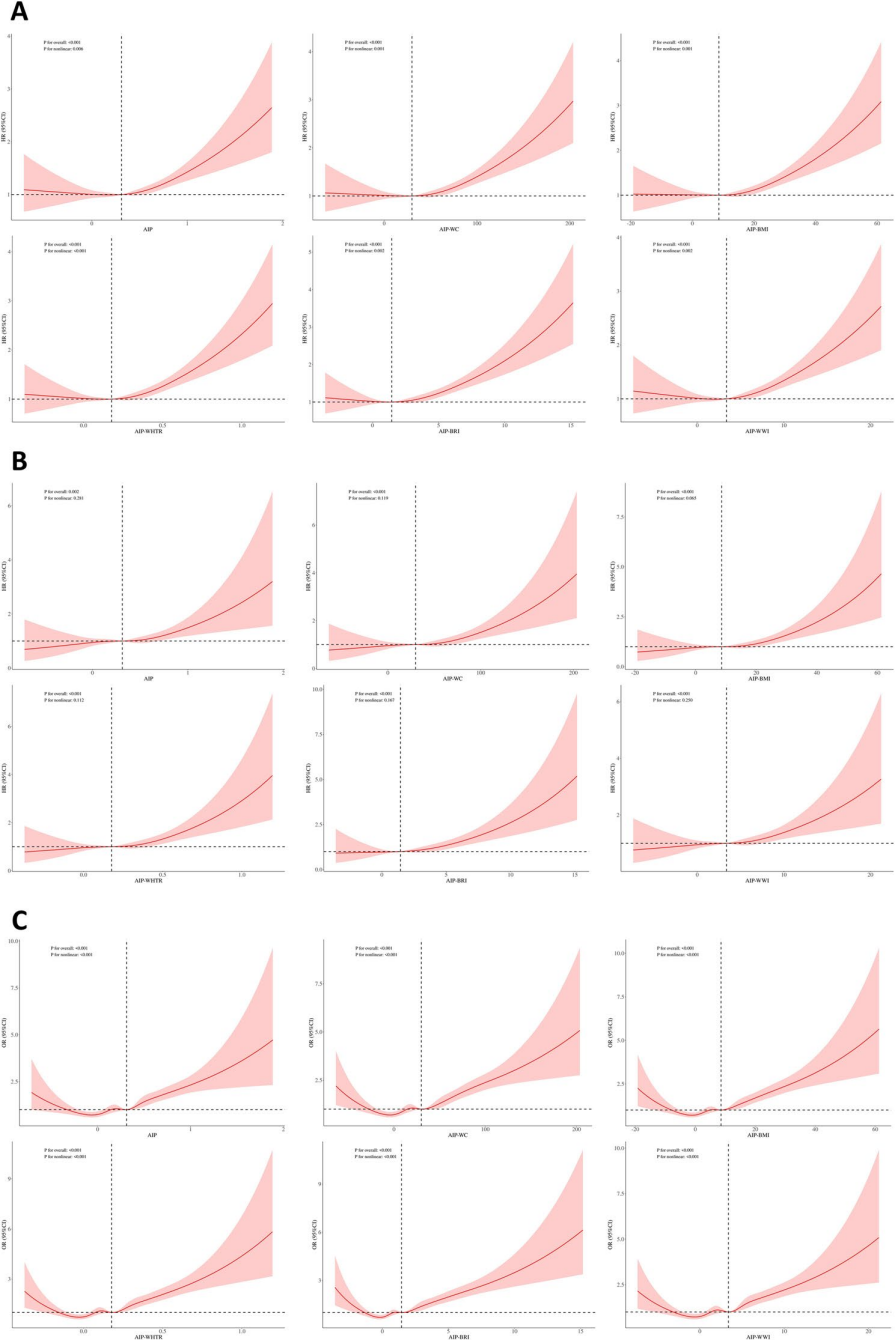
RCS curves showing the associations of AIP and AIP-obesity indices with all-cause mortality (**A**), CVD mortality (**B**), and CVD prevalence (**C**). Adjusted for age, sex, race, marital status, education, smoking status, alcohol use, income-to-poverty ratio, and total cholesterol

Figure 2 presents RCS curves illustrating the dose–response relationships of AIP and AIP-obesity indices with all-cause mortality, CVD mortality, and CVD prevalence. In the fully adjusted model (Model 3), AIP, AIP-WC, AIP-BMI, AIP-WHtR, AIP-WWI, and AIP-BRI demonstrated significant nonlinear associations with all-cause mortality and CVD prevalence (*P* for nonlinear < 0.05), whereas their associations with CVD mortality were linear (*P* for nonlinear > 0.05).

### Subgroup analyses

Subgroup analyses were performed according to age, sex, hypertension, and diabetes (Tables S5 and S6). No significant interactions were observed for all-cause mortality, CVD mortality, or CVD prevalence with AIP and AIP-obesity indices (AIP-WC, AIP-BMI, AIP-WHtR, AIP-WWI, and AIP-BRI) across subgroups of sex, hypertension, or diabetes (*P*-interaction > 0.05). In contrast, significant interactions were detected in age subgroups for the associations of AIP, AIP-WC, AIP-BMI, AIP-WHtR, AIP-WWI, and AIP-BRI with all-cause and CVD mortality (*P*-interaction < 0.05).

### Incremental predictive value analyses

Table 4 presents the incremental predictive value of AIP and AIP-obesity indices for CVD and mortality. After incorporating AIP and AIP-obesity indices into the baseline model, the concordance statistic (C-statistic), net reclassification improvement (NRI), and integrated discrimination improvement (IDI) were all improved. Compared with AIP alone, AIP-WC, AIP-BMI, AIP-WHtR, AIP-WWI, and AIP-BRI demonstrated superior risk reclassification and discriminative performance across all outcomes. Notably, among these indices, AIP-BRI showed the most prominent performance, exhibiting the highest overall incremental value.

**Table 4:** Incremental predictive value of AIP and AIP-obesity indices for all-cause mortality, CVD mortality, and CVD prevalence

Model	C-statistic (95%CI)	NRI (95% CI)	IDI (95% CI)
All-cause mortality			
Basic model	0.8545 (0.8483, 0.8604)	Reference	Reference
Basic model + AIP	0.8549 (0.8482, 0.8619)	0.1990 (0.1547, 0.2463)	0.0204 (0.0081, 0.0371)
Basic model + AIP-WC	0.8551 (0.8493, 0.8611)	0.2220 (0.1665, 0.2692)	0.0250 (0.0130, 0.0421)
Basic model + AIP-BMI	0.8550 (0.8495, 0.8610)	0.2727 (0.2250, 0.3159)	0.0242 (0.0105, 0.0406)
Basic model + AIP-WHTR	0.8551 (0.8490, 0.8610)	0.2147 (0.1615, 0.2671)	0.0246 (0.0128, 0.0405)
Basic model + AIP-BRI	0.8554 (0.8493, 0.8609)	0.2455 (0.1908, 0.3039)	0.0286 (0.0137, 0.0460)
Basic model + AIP-WWI	0.8551 (0.8483, 0.8614)	0.2063 (0.1582, 0.2498)	0.0226 (0.0096, 0.0386)
CVD mortality			
Basic model	0.8776 (0.8671, 0.8882)	Reference	Reference
Basic model + AIP	0.8787 (0.8689, 0.8895)	0.2235 (0.1611, 0.3026)	0.0314 (0.0078, 0.0696)
Basic model + AIP-WC	0.8790 (0.8702, 0.8888)	0.2661 (0.1772, 0.3285)	0.0410 (0.0167, 0.0741)
Basic model + AIP-BMI	0.8791 (0.8716, 0.8885)	0.3139 (0.2417, 0.3790)	0.0449 (0.0187, 0.0821)
Basic model + AIP-WHTR	0.8791 (0.8696, 0.8891)	0.2466 (0.1734, 0.3227)	0.0408 (0.0140, 0.0758)
Basic model + AIP-BRI	0.8796 (0.8706, 0.8891)	0.2834 (0.2045, 0.3782)	0.0497 (0.0236, 0.0899)
Basic model + AIP-WWI	0.8789 (0.8700, 0.8888)	0.2346 (0.1549, 0.3081)	0.0341 (0.0108, 0.0625)
CVD			
Basic model	0.8203 (0.8126, 0.8284)	Reference	Reference
Basic model + AIP	0.8267 (0.8207, 0.8352)	0.3021 (0.2552, 0.3409)	0.0089 (0.0060, 0.0129)
Basic model + AIP-WC	0.8282 (0.8197, 0.8364)	0.3273 (0.2784, 0.3806)	0.0107 (0.0073, 0.0146)
Basic model + AIP-BMI	0.8283 (0.8223, 0.8370)	0.3296 (0.2855, 0.3743)	0.0112 (0.0077, 0.0153)
Basic model + AIP-WHTR	0.8282 (0.8208, 0.8370)	0.3306 (0.2854, 0.3759)	0.0110 (0.0075, 0.0143)
Basic model + AIP-BRI	0.8292 (0.8223, 0.8371)	0.3617 (0.3134, 0.4096)	0.0123 (0.0087, 0.0159)
Basic model + AIP-WWI	0.8273 (0.8195, 0.8347)	0.3145 (0.2699, 0.3637)	0.0097 (0.0066, 0.0143)

The basic model included: age, sex, race, marital status, education, smoking status, alcohol use, income-to-poverty ratio, and total cholesterol

*Abbreviations*: *C-statistic* Concordance statistic, *NRI* Net reclassification improvement, *IDI* Integrated discrimination improvement, *CI* Confidence interval, *CVD* Cardiovascular disease, *AIP* Atherogenic index of plasma, *BMI* Body mass index, *WC* Waist circumference, *WHtR* Waist-to-height ratio, *BRI* Body roundness index, *WWI* Weight-adjusted waist index

### Sensitivity analyses

To assess the robustness of the findings, three sensitivity analyses were performed: (1) excluding participants who died during the initial two-year follow-up, (2) excluding those with a history of CVD or cancer, and (3) excluding participants with missing data. These analyses yielded results aligned with primary findings, validating the reliability of the findings (Tables S7–S11).

## Discussion

To our knowledge, this is the first study to evaluate the associations of AIP-obesity indices (AIP-WC, AIP-BMI, AIP-WHtR, AIP-WWI, and AIP-BRI) with all-cause and CVD mortality. Our findings indicate that AIP and AIP-obesity indices were significantly associated with all-cause mortality, CVD mortality, and CVD prevalence, additionally highlighting the incremental assessment value of the AIP-obesity indices.

Evidence demonstrates a positive correlation of raised AIP measurements with the occurrence of major adverse cardiovascular events [27]. Furthermore, evidence from a systematic review with meta-analysis indicates that higher AIP levels are associated with an increased risk of myocardial infarction, stent thrombosis, no-reflow events, revascularization, and CVD mortality [10]. Notably, studies have linked longitudinal changes in AIP to the risk of CVD-related mortality. Based on an analysis of 12-year follow-up data from 3,976 participants, Li et al. [28] reported that individuals with persistently high AIP levels had an approximately 33% increased risk of CVD-related mortality compared with their counterparts with persistently low levels. These results highlight the vital function of AIP in CVD risk evaluation.

In addition to atherosclerosis, obesity is recognized as an important metabolic factor influencing CVD [29, 30]. Growing evidence suggests that combining obesity-related indicators allows for a better evaluation of the risk of CVD and mortality [18, 23, 25, 31, 32]. Wang et al. [23], using data from 3,697 adults with cardiovascular-kidney-metabolic syndrome stages 0–3 in the China Health and Retirement Longitudinal Study (CHARLS), examined the associations of AIP, AIP-WC, AIP-WHtR, and AIP-BMI with stroke risk. Their findings indicated that integrating AIP with obesity-related indices yielded stronger risk stratification for stroke. Similarly, Zhang et al. [18] analyzed 4,519 middle-aged and older participants from CHARLS to investigate the cumulative changes in AIP, AIP-BMI, AIP-WC, AIP-WHtR, AIP-BRI, and AIP-CVAI in relation to CVD risk. The study showed that AIP combined with obesity indices provided improved CVD risk assessment compared with AIP alone. Compared with previous research, our work is the first to evaluate the relationships of AIP-obesity indices with both all-cause and CVD mortality. We found that, in assessing the risks of all-cause mortality, CVD mortality, and CVD prevalence in adults, AIP-WC, AIP-BMI, AIP-WHtR, AIP-WWI, and AIP-BRI provided superior risk stratification compared with AIP alone. Our findings provide new insights into this field and underscore the importance of jointly considering atherosclerosis and obesity in evaluating mortality risk and CVD. Given the easy accessibility of both AIP and obesity indicators, their combined application in clinical practice should be promoted, particularly among populations at high risk of CVD. Managing atherogenic dyslipidemia (AIP) and obesity should both be regarded as core strategies for reducing CVD risk in such individuals.

We conducted subgroup analyses and observed significant interactions between age and the associations of AIP, AIP-WC, AIP-BMI, AIP-WHtR, AIP-WWI, and AIP-BRI with all-cause and CVD mortality (*P*-interaction < 0.05). Among participants younger than 65 years, these indices exhibited stronger associations. We speculate that this interaction may be related to the trend of earlier onset of dyslipidemia [33, 34]. Dyslipidemia represents a well-recognized risk factor for CVD development and all-cause mortality, and individuals with early-onset lipid abnormalities generally face higher cardiovascular risk than those with later-onset abnormalities [33, 35]. The younger onset of dyslipidemia may strengthen the associations of AIP and AIP-obesity indices with mortality risk in this subgroup. Moreover, the observed age interaction may also reflect fewer confounding factors in younger populations. With generally higher metabolic activity and fewer comorbidities, dyslipidemia is a more direct determinant of CVD risk in this group [7]. Conversely, in older adults, the coexistence of multimorbidity may weaken this relationship. Furthermore, nonlinear associations were observed between AIP, AIP-WC, AIP-BMI, AIP-WHtR, AIP-WWI, AIP-BRI and all-cause mortality, whereas their associations with CVD mortality exhibited linear patterns in our study. CVD mortality is predominantly attributable to atherosclerotic CVD [1, 36]. Both elevated AIP and obesity have been established as significant risk factors for atherosclerosis [6, 13]. In contrast, all-cause mortality is predominantly composed of causes such as CVD, cancer, and respiratory diseases [37]. CVD deaths accounted for only 31.33% of all-cause deaths in this study population. These factors may collectively explain the observed discrepancy in the shapes of the RCS curves to some extent.

The use of data from 21,944 participants drawn from the nationally representative NHANES cohort, with a relatively long follow-up period, constitutes a major strength of this study. Methodologically, we employed a series of statistical approaches, including Cox and logistic regression modeling, RCS analysis, subgroup analysis, incremental predictive value assessment, and sensitivity analyses, thereby enhancing the reliability and rigor of our findings. Nevertheless, the interpretation of our findings must consider several study limitations. First, as the study cohort comprised mainly adults from the United States, its findings may have limited generalizability to other populations. Second, although multiple confounding factors were adjusted for, residual confounding cannot be completely excluded. Third, information on CVD was self-reported, which may introduce false positives. Notably, previous studies have demonstrated the high reliability of self-reported CVD events, which may mitigate the impact of this potential bias [38]. Finally, a portion of participants were excluded for not meeting the inclusion criteria, which may introduce selection bias.

## Conclusion

AIP, AIP-WC, AIP-BMI, AIP-WHtR, AIP-WWI, and AIP-BRI were significantly associated with all-cause mortality, CVD mortality, and CVD prevalence. The associations between these indices and mortality were stronger among participants younger than 65 years. AIP combined with obesity-related indices, particularly BRI, provides additional value for the stratification of CVD and mortality risk.

## Supplementary Information

Supplementary Material 1.

## Data Availability

The datasets analyzed in this study are obtainable from the corresponding author upon reasonable request.

## Abbreviations

AIP: Atherogenic index of plasma
CVD: Cardiovascular disease
WC: Waist circumference
BMI: Body mass index
WHtR: Waist-to-height ratio
BRI: Body roundness index
WWI: Weight-adjusted waist index
NHANES: National Health and Nutrition Examination Survey
TyG: Triglyceride-glucose index
RCS: Restricted cubic splines
PIR: Poverty-to-income ratio
HDL-C: High-density lipoprotein cholesterol
TG: Triglycerides
TC: Total cholesterol
HbA1c: Glycated hemoglobin
FBG: Fasting blood glucose
VIF: Variance inflation factor
SE: Standard error
HR: Hazard ratio
OR: Odds ratio
C-statistic: Concordance statistic
NRI: Net reclassification improvement
IDI: Integrated discrimination improvement
ELSA: English Longitudinal Study of Ageing
CHARLS: China Health and Retirement Longitudinal Study
CVAI: Chinese visceral adiposity index
