# Supplementary material for: Association of the atherogenic index of plasma combined with obesity indices with cardiovascular disease and mortality
Source: Lipids Health Dis. 2026 Mar 19;25:121. doi: 10.1186/s12944-026-02932-3 (PMC13122940; doi:10.1186/s12944-026-02932-3)
Supplement: Supplementary file 1 — Supplementary Material 1. [file 12944_2026_2932_MOESM1_ESM.docx]

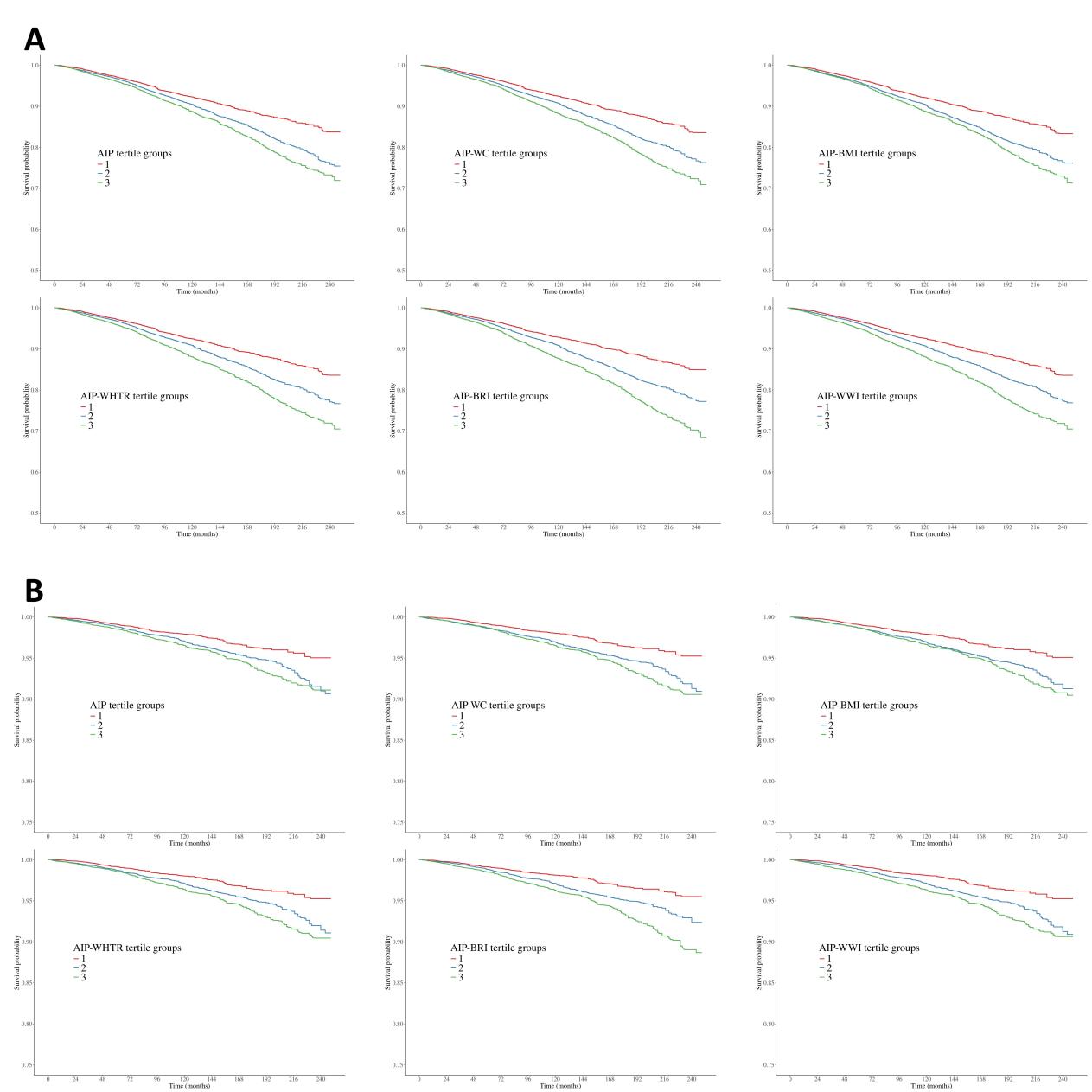


**Figure S1**. Kaplan–Meier survival curves for all-cause mortality (A) and CVD mortality (B) according to tertiles of AIP and AIP-obesity indices.

**Table S1. Baseline characteristics according to all-cause mortality.**

| Variable | Total  (n = 21944) | All-cause mortality | | *P* |
| --- | --- | --- | --- | --- |
|  |  | No (n=18618) | Yes (n=3326) |  |
| Sex, n (%) |  |  |  | **<0.001** |
| Male | 10922 (49.42) | 9035 (48.92) | 1887 (53.53) |  |
| Female | 11022 (50.58) | 9583 (51.08) | 1439 (46.47) |  |
| Age, year | 47.39 (0.22) | 45.10 (0.21) | 65.88 (0.41) | **<0.001** |
| Race, n (%) |  |  |  | **<0.001** |
| Mexican American | 3886 (8.18) | 3434 (8.73) | 452 (3.73) |  |
| Non-Hispanic White | 9820 (69.11) | 7792 (67.85) | 2028 (79.27) |  |
| Non-Hispanic Black | 4362 (10.42) | 3766 (10.50) | 596 (9.81) |  |
| Other Race | 3876 (12.29) | 3626 (12.92) | 250 (7.19) |  |
| Marital status, n (%) |  |  |  | **<0.001** |
| Married or living with partner | 11733 (52.74) | 10420 (54.63) | 1313 (37.58) |  |
| Separated/divorced/widowed | 5566 (25.71) | 4268 (24.21) | 1298 (37.79) |  |
| Never | 4645 (21.55) | 3930 (21.17) | 715 (24.62) |  |
| Education, n (%) |  |  |  | **<0.001** |
| Below high school | 5976 (17.59) | 4676 (16.13) | 1300 (29.40) |  |
| High school or equivalent | 5037 (24.08) | 4194 (23.48) | 843 (28.93) |  |
| College or above | 10931 (58.32) | 9748 (60.39) | 1183 (41.67) |  |
| Smoking status, n (%) |  |  |  | **<0.001** |
| Never | 11733 (52.74) | 10420 (54.63) | 1313 (37.58) |  |
| Former | 5566 (25.71) | 4268 (24.21) | 1298 (37.79) |  |
| Current | 4645 (21.55) | 3930 (21.17) | 715 (24.62) |  |
| Alcohol use, n (%) |  |  |  | **<0.001** |
| Never | 6472 (25.27) | 5356 (24.54) | 1116 (31.17) |  |
| Ever | 15472 (74.73) | 13262 (75.46) | 2210 (68.83) |  |
| PIR, n (%) |  |  |  | **<0.001** |
| < 1.3 | 6633 (21.07) | 5511 (20.48) | 1122 (25.83) |  |
| 1.3–3.5 | 8466 (36.50) | 7021 (35.47) | 1445 (44.77) |  |
| > 3.5 | 6845 (42.43) | 6086 (44.04) | 759 (29.40) |  |
| Height, cm | 169.02 (0.09) | 169.21 (0.10) | 167.48 (0.25) | **<0.001** |
| BMI, kg/m^2^ | 28.68 (0.08) | 28.70 (0.08) | 28.47 (0.16) | 0.185 |
| WC, cm | 98.39 (0.20) | 98.02 (0.21) | 101.38 (0.39) | **<0.001** |
| Hypertension, n (%) |  |  |  | **<0.001** |
| No | 12605 (62.80) | 11624 (66.47) | 981 (33.22) |  |
| Yes | 9339 (37.20) | 6994 (33.53) | 2345 (66.78) |  |
| Diabetes, n (%) |  |  |  | **<0.001** |
| No | 17695 (85.62) | 15514 (87.67) | 2181 (69.11) |  |
| Yes | 4249 (14.38) | 3104 (12.33) | 1145 (30.89) |  |
| FPG, mg/dL | 105.38 (0.30) | 103.84 (0.29) | 117.79 (1.00) | **<0.001** |
| HbA1c, % | 5.58 (0.01) | 5.54 (0.01) | 5.95 (0.03) | **<0.001** |
| TC, mg/dL | 195.38 (0.46) | 194.97 (0.47) | 198.63 (0.88) | **<0.001** |
| TG, mg/dL | 132.38 (1.22) | 129.65 (1.33) | 154.37 (2.11) | **<0.001** |
| HDL-C, mg/dL | 53.61 (0.19) | 53.63 (0.20) | 53.39 (0.43) | 0.588 |
| AIP | 0.33 (0.00) | 0.32 (0.00) | 0.41 (0.01) | **<0.001** |
| AIP-WC | 34.24 (0.43) | 33.06 (0.46) | 43.73 (0.81) | **<0.001** |
| AIP-BMI | 10.04 (0.13) | 9.75 (0.14) | 12.38 (0.24) | **<0.001** |
| AIP-WHTR | 0.20 (0.00) | 0.19 (0.00) | 0.26 (0.00) | **<0.001** |
| AIP-BRI | 1.94 (0.03) | 1.87 (0.03) | 2.57 (0.06) | **<0.001** |
| AIP-WWI | 3.64 (0.04) | 3.50 (0.05) | 4.77 (0.08) | **<0.001** |
| AIP tertile groups, n (%) |  |  |  | **<0.001** |
| 1 | 7101 (32.98) | 6334 (34.32) | 767 (22.18) |  |
| 2 | 7474 (33.97) | 6278 (33.84) | 1196 (34.99) |  |
| 3 | 7369 (33.05) | 6006 (31.83) | 1363 (42.84) |  |
| AIP-WC tertile groups, n (%) |  |  |  | **<0.001** |
| 1 | 7070 (33.00) | 6314 (34.33) | 756 (22.22) |  |
| 2 | 7549 (34.00) | 6356 (33.92) | 1193 (34.63) |  |
| 3 | 7325 (33.00) | 5948 (31.74) | 1377 (43.14) |  |
| AIP-BMI tertile groups, n (%) |  |  |  | **<0.001** |
| 1 | 7059 (33.00) | 6282 (34.26) | 777 (22.83) |  |
| 2 | 7549 (34.00) | 6304 (33.76) | 1245 (35.89) |  |
| 3 | 7336 (33.00) | 6032 (31.97) | 1304 (41.28) |  |
| AIP-WHTR tertile groups, n (%) |  |  |  | **<0.001** |
| 1 | 7050 (32.99) | 6301 (34.34) | 749 (22.11) |  |
| 2 | 7472 (34.00) | 6292 (33.98) | 1180 (34.19) |  |
| 3 | 7422 (33.00) | 6025 (31.67) | 1397 (43.70) |  |
| AIP-BRI tertile groups, n (%) |  |  |  | **<0.001** |
| 1 | 6960 (32.99) | 6247 (34.45) | 713 (21.29) |  |
| 2 | 7423 (34.00) | 6247 (33.92) | 1176 (34.65) |  |
| 3 | 7561 (33.00) | 6124 (31.63) | 1437 (44.06) |  |
| AIP-WWI tertile groups, n (%) |  |  |  | **<0.001** |
| 1 | 7069 (33.00) | 6326 (34.39) | 743 (21.76) |  |
| 2 | 7428 (34.00) | 6282 (34.03) | 1146 (33.72) |  |
| 3 | 7447 (33.00) | 6010 (31.58) | 1437 (44.51) |  |

Continuous variables were expressed as mean (SE), and group differences were assessed using the t-test or Mann-Whitney U test. Categorical variables were expressed as counts (percentages), and group differences were assessed using the chi-square test. Bold values indicate p-values < 0.05.

Abbreviations: PIR income-to-poverty ratio, BMI body mass index, WC waist circumference, FBG fasting blood glucose, HbA1c glycated hemoglobin, TC total cholesterol, TG triglycerides, HDL-C high-density lipoprotein cholesterol, AIP atherogenic index of plasma, WHtR waist-to-height ratio, BRI body roundness index, WWI weight-adjusted waist index, SE standard errors.

**Table S2. Baseline characteristics according to CVD.**

| Variable | Total  (n = 21944) | CVD | | *P* |
| --- | --- | --- | --- | --- |
|  |  | No (n=19536) | Yes (n=2408) |  |
| Sex, n (%) |  |  |  | **<0.001** |
| Male | 10922 (49.42) | 9555 (48.88) | 1367 (55.22) |  |
| Female | 11022 (50.58) | 9981 (51.12) | 1041 (44.78) |  |
| Age, year | 47.39 (0.22) | 45.79 (0.21) | 64.33 (0.37) | **<0.001** |
| Race, n (%) |  |  |  | **<0.001** |
| Mexican American | 3886 (8.18) | 3603 (8.55) | 283 (4.25) |  |
| Non-Hispanic White | 9820 (69.11) | 8490 (68.55) | 1330 (75.11) |  |
| Non-Hispanic Black | 4362 (10.42) | 3873 (10.40) | 489 (10.62) |  |
| Other Race | 3876 (12.29) | 3570 (12.50) | 306 (10.03) |  |
| Marital status, n (%) |  |  |  | **<0.001** |
| Married or living with partner | 13418 (64.96) | 12008 (64.98) | 1410 (64.71) |  |
| Separated/divorced/widowed | 4803 (18.19) | 3953 (17.03) | 850 (30.49) |  |
| Never | 3723 (16.85) | 3575 (17.99) | 148 (4.80) |  |
| Education, n (%) |  |  |  | **<0.001** |
| Below high school | 5976 (17.59) | 5094 (16.68) | 882 (27.23) |  |
| High school or equivalent | 5037 (24.08) | 4470 (23.85) | 567 (26.57) |  |
| College or above | 10931 (58.32) | 9972 (59.47) | 959 (46.20) |  |
| Smoking status, n (%) |  |  |  | **<0.001** |
| Never | 11733 (52.74) | 10800 (54.27) | 933 (36.62) |  |
| Former | 5566 (25.71) | 4603 (24.31) | 963 (40.49) |  |
| Current | 4645 (21.55) | 4133 (21.42) | 512 (22.88) |  |
| Alcohol use, n (%) |  |  |  | **<0.001** |
| Never | 6472 (25.27) | 5674 (24.84) | 798 (29.82) |  |
| Ever | 15472 (74.73) | 13862 (75.16) | 1610 (70.18) |  |
| PIR, n (%) |  |  |  | **<0.001** |
| < 1.3 | 6633 (21.07) | 5777 (20.51) | 856 (27.00) |  |
| 1.3–3.5 | 8466 (36.50) | 7474 (36.00) | 992 (41.78) |  |
| > 3.5 | 6845 (42.43) | 6285 (43.49) | 560 (31.22) |  |
| Height, cm | 169.02 (0.09) | 169.14 (0.10) | 167.72 (0.25) | **<0.001** |
| BMI, kg/m^2^ | 28.68 (0.08) | 28.55 (0.08) | 30.04 (0.20) | **<0.001** |
| WC, cm | 98.39 (0.20) | 97.75 (0.21) | 105.17 (0.47) | **<0.001** |
| Hypertension, n (%) |  |  |  | **<0.001** |
| No | 12605 (62.80) | 12056 (66.25) | 549 (26.38) |  |
| Yes | 9339 (37.20) | 7480 (33.75) | 1859 (73.62) |  |
| Diabetes, n (%) |  |  |  | **<0.001** |
| No | 17695 (85.62) | 16320 (87.86) | 1375 (61.95) |  |
| Yes | 4249 (14.38) | 3216 (12.14) | 1033 (38.05) |  |
| FPG, mg/dL | 105.38 (0.30) | 104.01 (0.29) | 119.83 (1.13) | **<0.001** |
| HbA1c, % | 5.58 (0.01) | 5.53 (0.01) | 6.08 (0.03) | **<0.001** |
| TC, mg/dL | 195.38 (0.46) | 196.35 (0.48) | 185.09 (1.26) | **<0.001** |
| TG, mg/dL | 132.38 (1.22) | 130.72 (1.25) | 149.96 (3.09) | **<0.001** |
| HDL-C, mg/dL | 53.61 (0.19) | 53.84 (0.19) | 51.10 (0.52) | **<0.001** |
| AIP | 0.33 (0.00) | 0.32 (0.00) | 0.41 (0.01) | **<0.001** |
| AIP-WC | 34.24 (0.43) | 33.22 (0.42) | 45.05 (1.26) | **<0.001** |
| AIP-BMI | 10.04 (0.13) | 9.76 (0.13) | 12.94 (0.37) | **<0.001** |
| AIP-WHTR | 0.20 (0.00) | 0.20 (0.00) | 0.27 (0.01) | **<0.001** |
| AIP-BRI | 1.94 (0.03) | 1.87 (0.03) | 2.76 (0.09) | **<0.001** |
| AIP-WWI | 3.64 (0.04) | 3.53 (0.04) | 4.79 (0.13) | **<0.001** |
| AIP tertile groups, n (%) |  |  |  | **<0.001** |
| 1 | 7101 (32.98) | 6553 (33.99) | 548 (22.32) |  |
| 2 | 7474 (33.97) | 6646 (34.01) | 828 (33.50) |  |
| 3 | 7369 (33.05) | 6337 (31.99) | 1032 (44.19) |  |
| AIP-WC tertile groups, n (%) |  |  |  | **<0.001** |
| 1 | 7070 (33.00) | 6548 (34.08) | 522 (21.52) |  |
| 2 | 7549 (34.00) | 6744 (34.16) | 805 (32.28) |  |
| 3 | 7325 (33.00) | 6244 (31.75) | 1081 (46.19) |  |
| AIP-BMI tertile groups, n (%) |  |  |  | **<0.001** |
| 1 | 7059 (33.00) | 6530 (34.06) | 529 (21.84) |  |
| 2 | 7549 (34.00) | 6724 (34.10) | 825 (32.90) |  |
| 3 | 7336 (33.00) | 6282 (31.84) | 1054 (45.27) |  |
| AIP-WHTR tertile groups, n (%) |  |  |  | **<0.001** |
| 1 | 7050 (32.99) | 6534 (34.11) | 516 (21.22) |  |
| 2 | 7472 (34.00) | 6676 (34.22) | 796 (31.71) |  |
| 3 | 7422 (33.00) | 6326 (31.67) | 1096 (47.07) |  |
| AIP-BRI tertile groups, n (%) |  |  |  | **<0.001** |
| 1 | 6960 (32.99) | 6473 (34.21) | 487 (20.12) |  |
| 2 | 7423 (34.00) | 6667 (34.36) | 756 (30.21) |  |
| 3 | 7561 (33.00) | 6396 (31.42) | 1165 (49.67) |  |
| AIP-WWI tertile groups, n (%) |  |  |  | **<0.001** |
| 1 | 7069 (33.00) | 6546 (34.09) | 523 (21.46) |  |
| 2 | 7428 (34.00) | 6625 (34.12) | 803 (32.68) |  |
| 3 | 7447 (33.00) | 6365 (31.79) | 1082 (45.86) |  |

Continuous variables were expressed as mean (SE), and group differences were assessed using the t-test or Mann-Whitney U test. Categorical variables were expressed as counts (percentages), and group differences were assessed using the chi-square test. Bold values indicate p-values < 0.05.

Abbreviations: CVD cardiovascular disease, PIR income-to-poverty ratio, BMI body mass index, WC waist circumference, FBG fasting blood glucose, HbA1c glycated hemoglobin, TC total cholesterol, TG triglycerides, HDL-C high-density lipoprotein cholesterol, AIP atherogenic index of plasma, WHtR waist-to-height ratio, BRI body roundness index, WWI weight-adjusted waist index, SE standard errors.

**Table S3. Variance inflation factor (VIF) values among exposure variables and covariates.**

| Variables | Model | | | | | |
| --- | --- | --- | --- | --- | --- | --- |
|  | AIP | AIP-WC | AIP-BMI | AIP-WHTR | AIP-BRI | AIP-WWI |
| AIP | 1.197 | - | - | - | - | - |
| AIP-WC | - | 1.179 | - | - | - | - |
| AIP-BMI | - | - | 1.148 | - | - | - |
| AIP-WHTR | - | - | - | 1.172 | - | - |
| AIP-BRI | - | - | - | - | 1.124 | - |
| AIP-WWI | - | - | - | - | - | 1.195 |
| Sex | 1.421 | 1.421 | 1.420 | 1.423 | 1.425 | 1.424 |
| Race | 1.183 | 1.183 | 1.165 | 1.164 | 1.142 | 1.172 |
| Smoking status | 1.323 | 1.316 | 1.311 | 1.319 | 1.309 | 1.326 |
| Alcohol use | 1.306 | 1.305 | 1.303 | 1.304 | 1.302 | 1.306 |
| Education | 1.187 | 1.187 | 1.187 | 1.187 | 1.187 | 1.187 |
| Marital status | 1.351 | 1.350 | 1.350 | 1.351 | 1.351 | 1.352 |
| PIR | 1.390 | 1.390 | 1.391 | 1.390 | 1.389 | 1.390 |
| TC | 1.265 | 1.266 | 1.266 | 1.266 | 1.268 | 1.266 |

Abbreviations: VIF Variance inflation factor, PIR income-to-poverty ratio, TC total cholesterol, AIP atherogenic index of plasma, BMI body mass index, WC waist circumference, WHtR waist-to-height ratio, BRI body roundness index, WWI weight-adjusted waist index.

**Table S4. Proportional hazards assessment of AIP and AIP-obesity indices with mortality outcomes using Schoenfeld residuals.**

| Variables | *P* | |
| --- | --- | --- |
|  | **All-cause mortality** | **CVD mortality** |
| AIP | 0.313 | 0.604 |
| AIP-WC | 0.234 | 0.902 |
| AIP-BMI | 0.119 | 0.821 |
| AIP-WHTR | 0.252 | 0.756 |
| AIP-BRI | 0.237 | 0.962 |
| AIP-WWI | 0.398 | 0.489 |

Abbreviations: AIP atherogenic index of plasma, BMI body mass index, WC waist circumference, WHtR waist-to-height ratio, BRI body roundness index, WWI weight-adjusted waist index.

**Table S5. Subgroup analyses of the associations of AIP and AIP-obesity indices with all-cause mortality and CVD mortality.**

| Subgroup | HR (95%CI), Model3 | | | | | |
| --- | --- | --- | --- | --- | --- | --- |
|  | AIP | AIP-WC | AIP-BMI | AIP-WHTR | AIP-BRI | AIP-WWI |
| **All-cause mortality** | | | | | | |
| Sex | | | | | | |
| Male | 1.373 (1.129 - 1.671) | 1.004 (1.002 - 1.005) | 1.013 (1.006 - 1.019) | 1.873 (1.368 - 2.563) | 1.074 (1.043 - 1.106) | 1.032 (1.014 - 1.050) |
| Female | 1.358 (1.045 - 1.765) | 1.003 (1.001 - 1.006) | 1.010 (1.003 - 1.018) | 1.686 (1.151 - 2.468) | 1.051 (1.019 - 1.085) | 1.029 (1.006 - 1.052) |
| *P*-interaction | 0.941 | 0.752 | 0.542 | 0.658 | 0.295 | 0.844 |
| Age | | | | | | |
| < 65 years | 1.735 (1.323 - 2.275) | 1.006 (1.003 - 1.008) | 1.020 (1.012 - 1.028) | 2.755 (1.819 - 4.173) | 1.107 (1.069 - 1.145) | 1.055 (1.029 - 1.081) |
| ≥ 65 years | 1.152 (0.955 - 1.389) | 1.002 (1.000 - 1.003) | 1.005 (0.999 - 1.011) | 1.324 (0.993 - 1.766) | 1.033 (1.007 - 1.060) | 1.015 (0.999 - 1.032) |
| *P*-interaction | **0.004** | **0.002** | **0.001** | **0.001** | **0.001** | **0.003** |
| Hypertension | | | | | | |
| No | 1.559 (1.180 - 2.061) | 1.004 (1.002 - 1.007) | 1.016 (1.007 - 1.025) | 2.139 (1.368 - 3.345) | 1.077 (1.034 - 1.122) | 1.041 (1.015 - 1.067) |
| Yes | 1.153 (0.942 - 1.411) | 1.002 (1.000 - 1.004) | 1.005 (0.999 - 1.012) | 1.366 (0.995 - 1.874) | 1.039 (1.009 - 1.069) | 1.016 (0.999 - 1.035) |
| *P*-interaction | 0.098 | 0.128 | 0.122 | 0.130 | 0.185 | 0.120 |
| Diabetes | | | | | | |
| No | 1.216 (0.972 - 1.522) | 1.002 (1.000 - 1.004) | 1.007 (0.999 - 1.014) | 1.427 (1.000 - 2.037) | 1.039 (1.005 - 1.074) | 1.020 (1.000 - 1.040) |
| Yes | 1.069 (0.82 - 1.395) | 1.001 (0.999 - 1.003) | 1.003 (0.996 - 1.011) | 1.186 (0.811 - 1.734) | 1.023 (0.993 - 1.055) | 1.008 (0.986 - 1.031) |
| *P*-interaction | 0.763 | 0.709 | 0.551 | 0.725 | 0.721 | 0.842 |
| **CVD mortality** | | | | | | |
| Sex | | | | | | |
| Male | 1.577 (1.122 - 2.216) | 1.005 (1.002 - 1.008) | 1.019 (1.009 - 1.030) | 2.454 (1.447 - 4.163) | 1.103 (1.054 - 1.155) | 1.045 (1.014 - 1.076) |
| Female | 1.389 (0.895 - 2.157) | 1.004 (1.000 - 1.008) | 1.014 (1.001 - 1.027) | 1.956 (1.042 - 3.675) | 1.078 (1.023 - 1.135) | 1.033 (0.996 - 1.071) |
| *P*-interaction | 0.454 | 0.451 | 0.296 | 0.382 | 0.292 | 0.461 |
| Age | | | | | | |
| < 65 years | 2.208 (1.332 - 3.661) | 1.009 (1.005 - 1.013) | 1.032 (1.017 - 1.047) | 4.654 (2.188 - 9.901) | 1.168 (1.103 - 1.237) | 1.080 (1.033 - 1.129) |
| ≥ 65 years | 1.182 (0.844 - 1.654) | 1.002 (0.999 - 1.005) | 1.007 (0.996 - 1.018) | 1.439 (0.854 - 2.425) | 1.045 (0.998 - 1.095) | 1.018 (0.989 - 1.048) |
| *P*-interaction | **0.007** | **0.002** | **0.002** | **0.002** | **0.001** | **0.005** |
| Hypertension | | | | | | |
| No | 1.448 (0.788 - 2.664) | 1.005 (0.999 - 1.010) | 1.017 (0.997 - 1.037) | 2.067 (0.799 - 5.347) | 1.083 (0.995 - 1.179) | 1.034 (0.981 - 1.091) |
| Yes | 1.350 (0.988 - 1.845) | 1.003 (1.001 - 1.006) | 1.012 (1.002 - 1.022) | 1.824 (1.134 - 2.933) | 1.069 (1.026 - 1.114) | 1.030 (1.003 - 1.058) |
| *P*-interaction | 0.671 | 0.606 | 0.615 | 0.661 | 0.646 | 0.693 |
| Diabetes | | | | | | |
| No | 1.125 (0.783 - 1.618) | 1.002 (0.999 - 1.006) | 1.007 (0.995 - 1.019) | 1.416 (0.790 - 2.538) | 1.052 (0.996 - 1.111) | 1.015 (0.984 - 1.048) |
| Yes | 1.309 (0.893 - 1.920) | 1.003 (0.999 - 1.006) | 1.011 (1.000 - 1.022) | 1.575 (0.914 - 2.714) | 1.046 (0.998 - 1.095) | 1.022 (0.990 - 1.056) |
| *P*-interaction | 0.319 | 0.465 | 0.320 | 0.446 | 0.713 | 0.462 |

Subgroup analyses were conducted to examine the associations across sex, age and hypertension and diabetes subgroups.

Adjusted for age, sex, race, marital status, education, smoking status, alcohol use, income-to-poverty ratio, and total cholesterol. Bold values indicate p-values < 0.05.

Abbreviations: HR hazard ratio, CI confidence interval, CVD cardiovascular disease, AIP atherogenic index of plasma, BMI body mass index, WC waist circumference, WHtR waist-to-height ratio, BRI body roundness index, WWI weight-adjusted waist index.

**Table S6. Subgroup analyses of the associations of AIP and AIP-obesity indices with CVD prevalence.**

| Subgroup | OR (95%CI), Model3 | | | | | |
| --- | --- | --- | --- | --- | --- | --- |
|  | AIP | AIP-WC | AIP-BMI | AIP-WHTR | AIP-BRI | AIP-WWI |
| **CVD** | | | | | | |
| Sex | | | | | | |
| Male | 2.865 (2.052 - 4.001) | 1.010 (1.008 - 1.013) | 1.037 (1.028 - 1.047) | 6.175 (3.758 - 10.148) | 1.188 (1.141 - 1.237) | 1.101 (1.070 - 1.134) |
| Female | 2.270 (1.649 - 3.124) | 1.008 (1.005 - 1.011) | 1.028 (1.018 - 1.038) | 3.832 (2.366 - 6.205) | 1.128 (1.084 - 1.173) | 1.074 (1.046 - 1.104) |
| *P*-interaction | 0.678 | 0.640 | 0.443 | 0.387 | 0.152 | 0.474 |
| Age | | | | | | |
| < 65 years | 3.011 (2.261 - 4.008) | 1.011 (1.009 - 1.014) | 1.038 (1.030 - 1.046) | 6.615 (4.334 - 10.097) | 1.186 (1.145 - 1.228) | 1.107 (1.080 - 1.135) |
| ≥ 65 years | 1.953 (1.379 - 2.766) | 1.006 (1.003 - 1.010) | 1.023 (1.011 - 1.035) | 2.916 (1.682 - 5.056) | 1.102 (1.048 - 1.159) | 1.059 (1.029 - 1.091) |
| *P*-interaction | 0.139 | 0.104 | 0.132 | 0.052 | **0.043** | 0.052 |
| Hypertension | | | | | | |
| No | 2.637 (1.726 - 4.027) | 1.010 (1.006 - 1.014) | 1.035 (1.022 - 1.049) | 5.520 (2.893 - 10.533) | 1.179 (1.118 - 1.244) | 1.093 (1.053 - 1.134) |
| Yes | 2.035 (1.539 - 2.691) | 1.007 (1.004 - 1.009) | 1.023 (1.015 - 1.032) | 3.203 (2.120 - 4.839) | 1.11 (1.073 - 1.148) | 1.066 (1.041 - 1.091) |
| *P*-interaction | 0.627 | 0.346 | 0.360 | 0.345 | 0.135 | 0.455 |
| Diabetes | | | | | | |
| No | 2.054 (1.554 - 2.715) | 1.007 (1.005 - 1.010) | 1.025 (1.016 - 1.034) | 3.448 (2.234 - 5.321) | 1.127 (1.085 - 1.171) | 1.068 (1.042 - 1.095) |
| Yes | 2.235 (1.641 - 3.043) | 1.008 (1.005 - 1.011) | 1.027 (1.018 - 1.037) | 3.714 (2.370 - 5.820) | 1.119 (1.079 - 1.160) | 1.072 (1.045 - 1.101) |
| *P*-interaction | 0.147 | 0.210 | 0.148 | 0.234 | 0.588 | 0.264 |

Subgroup analyses were conducted to examine the associations across sex, age and hypertension and diabetes subgroups.

Adjusted for age, sex, race, marital status, education, smoking status, alcohol use, income-to-poverty ratio, and total cholesterol. Bold values indicate p-values < 0.05.

Abbreviations: OR odds ratio, CI confidence interval, CVD cardiovascular disease, AIP atherogenic index of plasma, BMI body mass index, WC waist circumference, WHtR waist-to-height ratio, BRI body roundness index, WWI weight-adjusted waist index.

**Table S7. Association of AIP and its obesity-related indices with all-cause mortality and CVD mortality, excluding participants who died within 2 years of follow-up.**

| Variables | Model1 | Model2 | Model3 |
| --- | --- | --- | --- |
|  | HR (95%CI) | HR (95%CI) | HR (95%CI) |
| **All-cause mortality** | | | |
| AIP | 1.701 (1.480 - 1.956) | 1.456 (1.238 - 1.713) | 1.342 (1.151 - 1.565) |
| AIP-WC | 1.006 (1.004 - 1.007) | 1.004 (1.002 - 1.005) | 1.003 (1.002 - 1.005) |
| AIP-BMI | 1.015 (1.011 - 1.019) | 1.013 (1.008 - 1.018) | 1.011 (1.006 - 1.016) |
| AIP-WHTR | 2.810 (2.257 - 3.498) | 1.965 (1.530 - 2.525) | 1.736 (1.375 - 2.191) |
| AIP-BRI | 1.113 (1.093 - 1.134) | 1.072 (1.049 - 1.097) | 1.060 (1.038 - 1.082) |
| AIP-WWI | 1.063 (1.049 - 1.077) | 1.037 (1.023 - 1.052) | 1.029 (1.015 - 1.043) |
| **CVD mortality** | | | |
| AIP | 1.676 (1.341 - 2.096) | 1.491 (1.129 - 1.969) | 1.418 (1.075 - 1.871) |
| AIP-WC | 1.006 (1.004 - 1.008) | 1.005 (1.002 - 1.007) | 1.004 (1.002 - 1.007) |
| AIP-BMI | 1.017 (1.010 - 1.024) | 1.018 (1.009 - 1.027) | 1.016 (1.008 - 1.025) |
| AIP-WHTR | 3.012 (2.109 - 4.301) | 2.287 (1.493 - 3.502) | 2.101 (1.381 - 3.196) |
| AIP-BRI | 1.130 (1.095 - 1.165) | 1.099 (1.058 - 1.141) | 1.088 (1.050 - 1.128) |
| AIP-WWI | 1.064 (1.042 - 1.085) | 1.040 (1.016 - 1.065) | 1.035 (1.011 - 1.060) |

Associations of AIP and AIP-obesity indices with all-cause and CVD mortality were assessed via Cox proportional hazards regression.

Model 1 was unadjusted; Model 2 was adjusted for age, sex, and race; Model 3 was adjusted for age, sex, race, marital status, education, smoking status, alcohol use, income-to-poverty ratio, and total cholesterol.

Abbreviations: HR hazard ratio, CI confidence interval, CVD cardiovascular disease, AIP atherogenic index of plasma, BMI body mass index, WC waist circumference, WHtR waist-to-height ratio, BRI body roundness index, WWI weight-adjusted waist index.

**Table S8. Association of AIP and its obesity-related indices with CVD prevalence, excluding participants who died within 2 years of follow-up.**

| Variables | Model1 | Model2 | Model3 |
| --- | --- | --- | --- |
|  | OR (95%CI) | OR (95%CI) | OR (95%CI) |
| **CVD** | | | |
| AIP | 2.302 (1.925 - 2.753) | 2.389 (1.927 - 2.960) | 2.692 (2.120 - 3.418) |
| AIP-WC | 1.009 (1.007 - 1.011) | 1.009 (1.007 - 1.011) | 1.010 (1.008 - 1.012) |
| AIP-BMI | 1.027 (1.022 - 1.033) | 1.032 (1.025 - 1.038) | 1.035 (1.028 - 1.042) |
| AIP-WHTR | 4.812 (3.622 - 6.394) | 4.716 (3.393 - 6.557) | 5.438 (3.829 - 7.723) |
| AIP-BRI | 1.171 (1.143 - 1.199) | 1.164 (1.132 - 1.196) | 1.170 (1.138 - 1.203) |
| AIP-WWI | 1.091 (1.074 - 1.109) | 1.085 (1.065 - 1.105) | 1.094 (1.072 - 1.116) |

Associations of AIP and AIP-obesity indices with CVD prevalence were assessed via logistic regression.

Model 1 was unadjusted; Model 2 was adjusted for age, sex, and race; Model 3 was adjusted for age, sex, race, marital status, education, smoking status, alcohol use, income-to-poverty ratio, and total cholesterol.

Abbreviations: OR odds ratio, CI confidence interval, CVD cardiovascular disease, AIP atherogenic index of plasma, BMI body mass index, WC waist circumference, WHtR waist-to-height ratio, BRI body roundness index, WWI weight-adjusted waist index.

**Table S9. Association of AIP and its obesity-related indices with all-cause mortality and CVD mortality, excluding participants with baseline CVD or cancer.**

| Variables | Model1 | Model2 | Model3 |
| --- | --- | --- | --- |
|  | HR (95%CI) | HR (95%CI) | HR (95%CI) |
| **All-cause mortality** | | | |
| AIP | 1.810 (1.496 - 2.189) | 1.551 (1.247 - 1.930) | 1.447 (1.164 - 1.799) |
| AIP-WC | 1.006 (1.004 - 1.008) | 1.004 (1.002 - 1.006) | 1.004 (1.002 - 1.006) |
| AIP-BMI | 1.017 (1.011 - 1.023) | 1.014 (1.008 - 1.021) | 1.013 (1.007 - 1.020) |
| AIP-WHTR | 3.107 (2.307 - 4.185) | 2.167 (1.550 - 3.030) | 1.958 (1.407 - 2.724) |
| AIP-BRI | 1.124 (1.096 - 1.153) | 1.083 (1.051 - 1.115) | 1.073 (1.042 - 1.105) |
| AIP-WWI | 1.069 (1.050 - 1.087) | 1.043 (1.024 - 1.064) | 1.036 (1.016 - 1.056) |
| **CVD mortality** | | | |
| AIP | 1.679 (1.233 - 2.285) | 1.469 (1.115 - 1.935) | 1.395 (1.068 - 1.823) |
| AIP-WC | 1.006 (1.003 - 1.009) | 1.005 (1.001 - 1.008) | 1.004 (1.001 - 1.008) |
| AIP-BMI | 1.018 (1.008 - 1.028) | 1.018 (1.006 - 1.030) | 1.017 (1.005 - 1.029) |
| AIP-WHTR | 3.070 (1.870 - 5.041) | 2.216 (1.261 - 3.895) | 2.065 (1.189 - 3.587) |
| AIP-BRI | 1.136 (1.088 - 1.186) | 1.101 (1.045 - 1.160) | 1.092 (1.038 - 1.150) |
| AIP-WWI | 1.063 (1.035 - 1.093) | 1.037 (1.006 - 1.070) | 1.032 (1.001 - 1.064) |

Associations of AIP and AIP-obesity indices with all-cause and CVD mortality were assessed via Cox proportional hazards regression.

Model 1 was unadjusted; Model 2 was adjusted for age, sex, and race; Model 3 was adjusted for age, sex, race, marital status, education, smoking status, alcohol use, income-to-poverty ratio, and total cholesterol.

Abbreviations: HR hazard ratio, CI confidence interval, CVD cardiovascular disease, AIP atherogenic index of plasma, BMI body mass index, WC waist circumference, WHtR waist-to-height ratio, BRI body roundness index, WWI weight-adjusted waist index.

**Table S10. Association of AIP and its obesity-related indices with all-cause mortality and CVD mortality, excluding participants with imputed covariates.**

| Variables | Model1 | Model2 | Model3 |
| --- | --- | --- | --- |
|  | HR (95%CI) | HR (95%CI) | HR (95%CI) |
| **All-cause mortality** | | | |
| AIP | 1.715 (1.475 - 1.994) | 1.492 (1.258 - 1.769) | 1.362 (1.164 - 1.593) |
| AIP-WC | 1.006 (1.004 - 1.007) | 1.004 (1.003 - 1.006) | 1.003 (1.002 - 1.005) |
| AIP-BMI | 1.015 (1.011 - 1.020) | 1.014 (1.009 - 1.019) | 1.012 (1.007 - 1.017) |
| AIP-WHTR | 2.881 (2.277 - 3.646) | 2.066 (1.588 - 2.689) | 1.792 (1.412 - 2.276) |
| AIP-BRI | 1.117 (1.096 - 1.139) | 1.078 (1.054 - 1.104) | 1.064 (1.042 - 1.087) |
| AIP-WWI | 1.064 (1.050 - 1.079) | 1.040 (1.025 - 1.056) | 1.030 (1.016 - 1.045) |
| **CVD mortality** | | | |
| AIP | 1.786 (1.413 - 2.258) | 1.597 (1.193 - 2.137) | 1.495 (1.127 - 1.985) |
| AIP-WC | 1.007 (1.004 - 1.009) | 1.005 (1.003 - 1.008) | 1.005 (1.002 - 1.007) |
| AIP-BMI | 1.018 (1.011 - 1.026) | 1.019 (1.010 - 1.028) | 1.018 (1.009 - 1.026) |
| AIP-WHTR | 3.296 (2.294 - 4.737) | 2.514 (1.627 - 3.884) | 2.255 (1.483 - 3.429) |
| AIP-BRI | 1.137 (1.103 - 1.171) | 1.106 (1.066 - 1.148) | 1.094 (1.056 - 1.133) |
| AIP-WWI | 1.070 (1.048 - 1.093) | 1.047 (1.021 - 1.074) | 1.040 (1.015 - 1.065) |

Associations of AIP and AIP-obesity indices with all-cause and CVD mortality were assessed via Cox proportional hazards regression.

Model 1 was unadjusted; Model 2 was adjusted for age, sex, and race; Model 3 was adjusted for age, sex, race, marital status, education, smoking status, alcohol use, income-to-poverty ratio, and total cholesterol.

Abbreviations: HR hazard ratio, CI confidence interval, CVD cardiovascular disease, AIP atherogenic index of plasma, BMI body mass index, WC waist circumference, WHtR waist-to-height ratio, BRI body roundness index, WWI weight-adjusted waist index.

**Table S11. Association of AIP and its obesity-related indices with CVD prevalence, excluding participants with imputed covariates.**

| Variables | Model1 | Model2 | Model3 |
| --- | --- | --- | --- |
|  | OR (95%CI) | OR (95%CI) | OR (95%CI) |
| **CVD** | | | |
| AIP | 2.257 (1.862 - 2.735) | 2.322 (1.856 - 2.904) | 2.628 (2.049 - 3.370) |
| AIP-WC | 1.009 (1.007 - 1.011) | 1.009 (1.007 - 1.011) | 1.010 (1.008 - 1.012) |
| AIP-BMI | 1.027 (1.020 - 1.033) | 1.031 (1.024 - 1.038) | 1.034 (1.027 - 1.042) |
| AIP-WHTR | 4.724 (3.460 - 6.449) | 4.545 (3.199 - 6.458) | 5.272 (3.622 - 7.674) |
| AIP-BRI | 1.170 (1.139 - 1.202) | 1.161 (1.126 - 1.197) | 1.168 (1.132 - 1.205) |
| AIP-WWI | 1.090 (1.071 - 1.110) | 1.082 (1.061 - 1.104) | 1.092 (1.069 - 1.115) |

Associations of AIP and AIP-obesity indices with CVD prevalence were assessed via logistic regression.

Model 1 was unadjusted; Model 2 was adjusted for age, sex, and race; Model 3 was adjusted for age, sex, race, marital status, education, smoking status, alcohol use, income-to-poverty ratio, and total cholesterol.

Abbreviations: OR odds ratio, CI confidence interval, CVD cardiovascular disease, AIP atherogenic index of plasma, BMI body mass index, WC waist circumference, WHtR waist-to-height ratio, BRI body roundness index, WWI weight-adjusted waist index.
